# Low-density lipoprotein cholesterol levels and in-hospital bleeding in patients with atrial fibrillation: findings from CCC-AF project

**DOI:** 10.3389/fcvm.2025.1574796

**Published:** 2025-06-12

**Authors:** Xue-ying Tong, Jing Lin, Zhao-qing Sun, Qian He, Yu Zhan, Chen-Xi Jiang, Ri-bo Tang, Cai-hua Sang, Man Ning, Chang-qi Jia, Li Feng, Wei Wang, Xin Zhao, Chang-yi Li, Song-nan Li, Xue-yuan Guo, Tong Liu, Meng-meng Li, Na Yang, Yong-chen Hao, Jun Liu, Jing Liu, Jin Xie, De-yong Long, Jian-zeng Dong, Dong Zhao, Chang-sheng Ma

**Affiliations:** ^1^ Department of Cardiology, Beijing Anzhen Hospital, Capital Medical University, Beijing, China; ^2^ Department of Geriatrics, Taihe Hospital, Hubei University of Medicine, Shiyan, China; ^3^ National Clinical Research Center for Cardiovascular Diseases, Beijing, China; ^4^ Department of Cardiology, Taihe Hospital, Hubei University of Medicine, Shiyan, China; ^5^ Department of Cardiology, Renmin Hospital, Hubei University of Medicine, Shiyan, China; ^6^ Department of Epidemiology, Beijing Anzhen Hospital, Capital Medical University, Beijing Institute of Heart, Lung and Blood Vessel Diseases, Beijing, China; ^7^ Hubei University of Medicine, Shiyan, China

**Keywords:** atrial fibrillation, low-density lipoprotein cholesterol, bleeding risk, nonlinear relationship, CCC-AF project

## Abstract

**Background and aims:**

Emerging evidence indicates a relationship between low-density lipoprotein cholesterol (LDL-C) levels and bleeding. However, data regarding the relationship between LDL-C levels and bleeding events in patients with atrial fibrillation (AF) remain unfilled. This study is aimed to examine the relationship between LDL-C levels and the risk of in-hospital bleeding in patients with AF.

**Methods and results:**

In this multi-centered observational study, 25,380 patients with AF were enrolled; 14,071 (55.4%) and 11 309 (44.6%) were men and women, respectively, and the mean age was 69.51 ± 11.88 years. After adjusting for covariates, with LDL-C ≥ 70 mg/dl as the reference, LDL-C < 70 mg/dl was associated with a higher risk of any bleeding event [adjusted odds ratio [aOR]: 1.63, 95% confidence interval [CI]: 1.12–2.35; *P* = 0.009], major bleeding events (aOR: 1.48, 95% CI: 0.99–2.20; *P* = 0.05), and gastrointestinal bleeding events (aOR: 2.11, 95% CI: 1.27–3.50; *P* = 0.004) in the multivariate logistic regression model. The restricted cubic spline model showed an L-shaped relationship for bleeding events, with a higher risk at lower LDL-C levels. The nonlinear relationship between LDL-C levels and the risk of bleeding persisted among the subgroups.

**Conclusions:**

This nationwide and multi-centered AF registry study found an L-shaped relationship between LDL-C levels at admission and in-hospital bleeding events, with a greater risk at lower LDL-C levels. Further studies are needed to establish LDL-C as a factor for risk stratification and management of bleeding events in patients with AF.

**Clinical Trial Registration:**

[http://www.clinicaltrials.gov], identifier [NCT02309398].

## Graphical Abstract

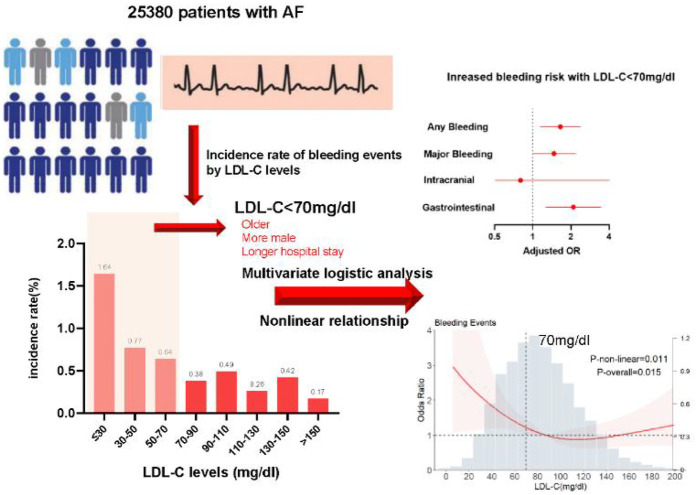

## Introduction

Atrial fibrillation (AF) is a common type of arrhythmia in the older adult population. Globally, 60 million (95% uncertainty interval: 32.5–42.6 million) individuals had AF or atrial flutter in 2022 (1), and 12 million were from China (2). AF is related to increased cardiovascular morbidity and mortality, and thus, it has a high global health and economic burden. Identifying patients with AF who are at the highest risk for bleeding events after anticoagulants is crucial for making informed clinical decisions to minimize bleeding complications. As such, biomarkers for predicting the bleeding risk have become important.

Low-density lipoprotein cholesterol (LDL-C) is a metabolic protein that regulates vascular disease through various mechanisms. It is well recognized as a risk factor for the development of atherosclerotic cardiovascular disease (ASCVD) events. High LDL-C levels consistently predict the risk of future ASCVD events in various populations worldwide (3). The current American Heart Association (AHA) guideline recommends that if a 10-year ASCVD risk ≥20% is estimated, a maximally tolerated statin dose should be used to lower LDL-C levels by >50% when the LDL-C is >70 mg/dl (4). A Korean study also found a unique relationship between LDL-C levels and mortality when lipid-lowering drugs were not used. This relationship followed a U-shaped pattern, indicating that higher and lower LDL-C levels were associated with an increased risk (5). Although emerging research has suggested that low levels of LDL-C are associated with bleeding (6–10), data regarding the relationship between low levels of LDL-C and bleeding events in patients with AF are limited.

Thus, this study aimed to investigate the relationship between LDL-C levels and the occurrence of in-hospital bleeding events in patients enrolled in the Improving Care for Cardiovascular Disease in China-Atrial Fibrillation (CCC-AF) project (11), which offered a substantial sample size to explore these relationships. We hypothesized that, similar to the results observed in randomized clinical trials, the LDL-C level is a biomarker for in-hospital bleeding events in individuals with AF.

## Methods

### Data collection and patients

The CCC-AF was launched in February 2015 as a cooperative project between the Chinese Society of Cardiology and AHA across 30 provinces in China. The methods and baseline data from the CCC-AF project, a national registry to improve AF management, have been previously reported (11). Patients with AF were included based on electrocardiograph (ECG) results recorded using a 12-lead ECG, 24 h Holter, or other rhythm monitors. The data elements and definitions of each variable were in accordance with the American College of Cardiology/AHA recommendations for AF clinical data standards (12, 13). The current study included patients with baseline LDL-C assessments from 236 hospitals that participated in the CCC-AF project between February 2015 and December 2019. The CCC-AF project was approved by the institutional review board of Beijing Anzhen Hospital, with a waiver for informed consent (number 2014018).

### Definitions

The data collected included patient demographics, medical history, and laboratory test results at admission. The CHA_2_DS_2_-VASc score was calculated according to age, congestive heart failure, hypertension, diabetes, stroke or transient ischemic attacks, vascular disease, and female sex (14). The HAS-BLED score was calculated according to uncontrolled hypertension, abnormal renal and/or hepatic function, stroke, bleeding history or predisposition, age >65 years, drugs, or excessive alcohol use (15). LDL-C values were obtained within 24 h of the first medical contact. Patients with LDL-C levels ≥70 mg/dl estimated to have a 7.5% higher risk for ASCVD within 10 years are recommended to start using moderate-intensity statin or maximally tolerated statin with 20% higher risk therapy in the current AHA guidelines (4). Thus, we estimated the relationship between LDL-C levels <70 mg/dl and study outcomes with LDL-C levels ≥70 mg/dl as the reference.

### Outcomes

An in-hospital bleeding event was defined as any occurrence of bleeding, including intracranial and gastrointestinal bleeding. Major bleeding events included fatal bleeding events, hemoglobin count decreasing to >20 g/L, total amount of transfusion >2U, or bleeding in vital organs or locations. Ischemic events included coronary heart disease, acute coronary syndrome, ischemic stroke, transient ischemic attack (TIA), pulmonary embolism, and peripheral arterial disease. All-cause deaths included in-hospital deaths regardless of the cause. The clinical outcome was defined as any occurrence of bleeding events, ischemic events, or all-cause death.

### Statistical analyses

Continuous variables were expressed as means and standard deviations or interquartile ranges. They were compared using the *t*-test if the data conformed to a normal distribution or the Wilcoxon rank-sum test if they did not. Categorical variables were compared using the appropriate chi-square test or Fisher's exact test. We conducted univariate and multivariate logistic analyses in four models to investigate the independent determinants of clinical patient status according to LDL-C levels as a continuous variable or as a categorical variable: (i) crude logistic regression models; (ii)multivariable-adjusted logistic regression models adjusted for age and sex;(iii) multivariable-adjusted logistic regression models adjusted for sex, age, tertiary hospital, hospital stay, smoking history, drinking history, medical history including Diabetes mellitus, hypertension, stroke or TIA, heart failure, chronic obstructive pulmonary disease (COPD), chronic liver disease, bleeding history, chronic kidney disease, anemia, cancer, left ventricular ejection fraction (LVEF) ≤40%, prior antiarrhythmic drugs, prior catheter ablation, prior surgical ablation, prior statins, prior antiplatelet, prior anticoagulant; (iv) multivariable-adjusted logistic regression models adjusted for variates in model 3 with 1,000 bootstrapping replications performed for internal validation. LDL-C levels were further stratified according to a stepwise increase of 20 mg/dl from <30 mg/dl to 150 mg/dl. Univariate logistic regression models were used to examine the incidence and relationship of bleeding risk with clinical outcomes.

The nonlinear relationship was evaluated using restricted cubic splines in logistic regression models, and the models were adjusted for covariates included in the multivariable logistic model unless otherwise specified. To test whether the nonlinear relationship between LDL-C level and bleeding outcomes was consistent across the established subgroups. The overall nonlinear *P*-value and interactive *P* values were calculated and presented. All statistical analyses were performed using SPSS (version 27) and R (version 4.3.2). A two-sided *P*-value of <0.05 was considered significant.

### Patient and public involvement statement

The public and patients have not been engaged in the research question proposal, design, recruitment, and implementation of the study. The results will be dispersed to study participants by public reporting.

## Results

### Patient characteristics

**Figure 1:**
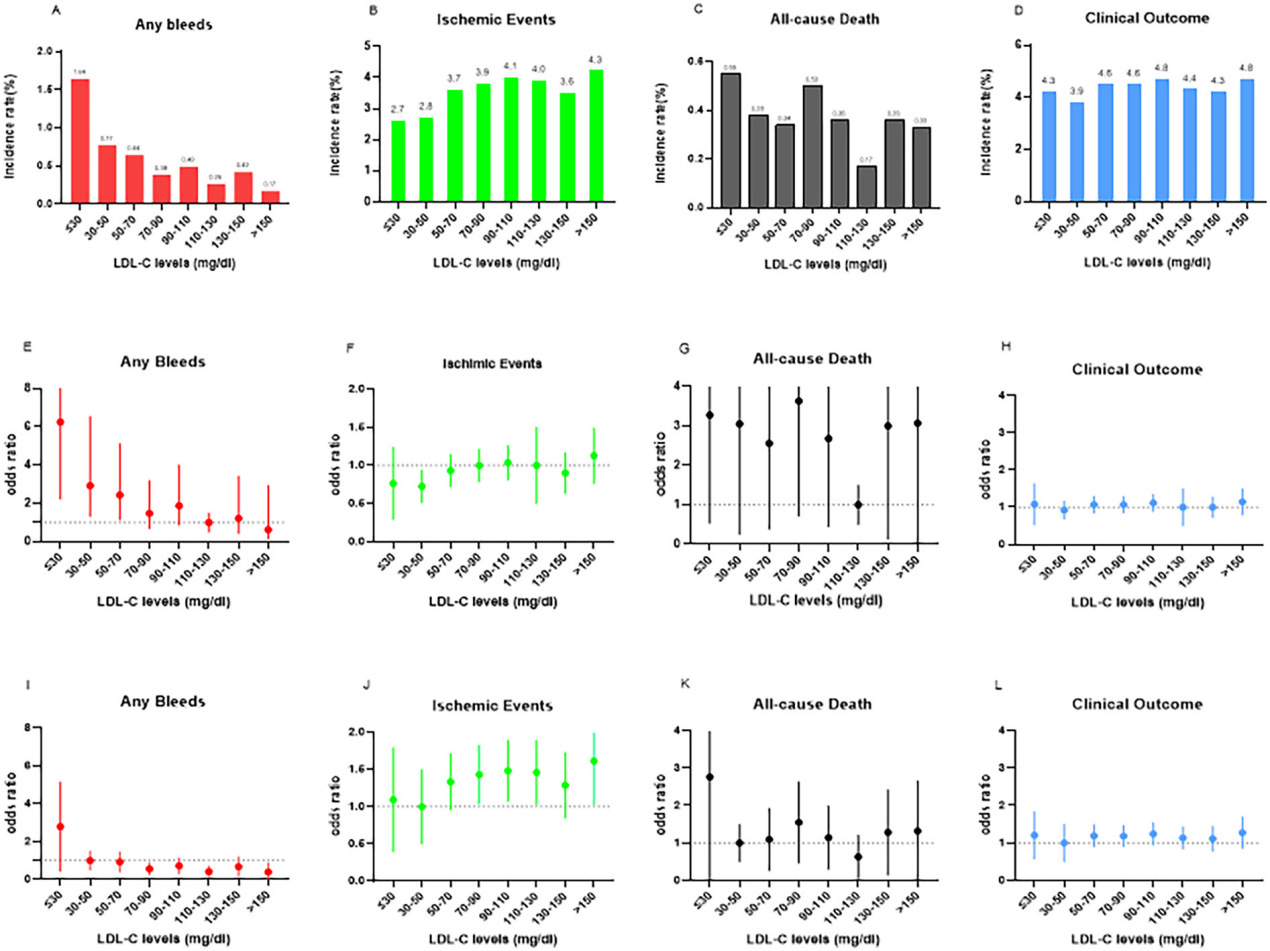
In-hospital bleeding events, ischemic events, all-cause death, and clinical outcomes according to the eight LDL-C level categories **(A–D)** In-hospital bleeding events, ischemic events, all-cause death and clinical outcomes according to the eight LDL-C categories. The numbers in parentheses indicate incidence in each LDL-C category. **(E–H)** Odds ratios (OR) and 95% confidence intervals (CIs) in the eight LDL-C level categories. The reference is set at LDL-C of 110-130 mg/dl in any bleeding event, ischemic events, clinical outcome, and all-cause death. **(I–L)** ORs and 95% CIs in the eight LDL-C level categories with LDL-C levels of 30-50 mg/dl as reference. LDL-C, low-density lipoprotein cholesterol.

In total, 25,380 patients were included. Among them, 14,071 (55.4%) and 11,309 (44.6%) patients were men and women, respectively, and the mean age was 69.51 ± 11.88 years. Bleeding events occurred more often in patients with LDL-C < 70 mg/dl, 54/7,449 (0.7%), than in patients with LDL-C < 70 mg/dl, 68/17,931(0.4%). The patient characteristics are shown in Table 1. Overall, 122 bleeding events, 93 all-cause deaths, and 959 ischemic events occurred within a median hospital stay of 8.77 days. The incidence rates of in-hospital bleeding, ischemic events, all-cause death, and clinical events according to eight LDL-C categories are presented in Figure 1.

**Table 1:** Clinical characteristics of patients stratified by the level of low-density lipoprotein cholesterol.

Characteristics	LDL ≥ 70 mg/dl	LDL < 70 mg/dl	Total population	*P*-value
Age, years, mean ± SD	68.63 (11.98)	71.62 (11.38)	69.5 (11.88)	<0.001
Age, years, *n* (%)				<0.001
≤64	6,181 (34.5)	1,787 (24.0)	7,968 (31.4)	
65–74	5,629 (31.4)	2,303 (30.9)	7,932 (31.3)	
≥75	6,121 (34.1)	3,359 (45.1)	9,480 (37.4)	
Age groups			0.005	
2015 and before	2 (0.0)	2 (0.0)	4 (0.0)	
2016	23 (0.1)	4 (0.1)	27 (0.1)	
2017	3,628 (20.3)	1,451 (19.5)	5,079 (20.0)	
2018	7,148 (39.9)	2,854 (38.4)	10,002 (39.5)	
2019 and after	7,114 (39.7)	3,120 (42.0)	10,234 (40.4)	
Length of stay (SD)	8.60 (5.41)	9.18 (5.77)	8.77 (5.52)	<0.001
Male, *n* (%)	9,780 (54.5)	4,291 (57.6)	14,071 (55.4)	<0.001
Han nationality, *n* (%)	17,400 (97.0)	7,245 (97.3)	24,645 (97.1)	0.335
Tertiary hospitals, *n* (%)	12,593 (70.2)	5,221 (70.1)	17,814 (70.2)	0.824
Medical insurance, *n* (%)				0.710
High cover	11,264 (62.8)	4,671 (62.7)	15,935 (62.8)	
Moderate cover	3,741 (20.9)	1,584 (21.3)	5,325 (21.0)	
Low cover	2,926 (16.3)	1,194 (16.0)	4,120 (16.2)	
Sinus rhythm, *n* (%)	3,949 (22.0)	1,325 (17.8)	5,274 (20.8)	<0.001
Medical history, *n* (%)				
Drinking	2,369 (13.2)	973 (13.1)	3,342 (13.2)	0.748
Smoking	3,870 (21.6)	1,585 (21.3)	5,455 (21.5)	0.590
Hypertension	9,778 (54.5)	4,246 (57.0)	14,024 (55.3)	<0.001
Diabetes mellitus	2,893 (16.1)	1,506 (20.2)	4,399 (17.3)	<0.001
CAD and PCI	4,778 (26.6)	2,513 (33.7)	7,291 (28.7)	<0.001
Stroke or TIA	2,268 (12.6)	1,251 (16.8)	3,519 (13.9)	<0.001
PAD	280 (1.6)	140 (1.9)	420 (1.7)	0.071
Heart failure	1,709 (9.5)	970 (13.0)	2,679 (10.6)	<0.001
Major bleed	261 (1.5)	146 (2.0)	407 (1.6)	0.004
COPD	797 (4.4)	477 (6.4)	1,274 (5.0)	<0.001
Prior MI	653 (3.6)	429 (5.8)	1,082 (4.3)	<0.001
Chronic liver disease	399 (2.2)	213 (2.9)	612 (2.4)	0.003
Chronic kidney disease	320 (1.8)	228 (3.1)	548 (2.2)	<0.001
Heart rate, bpm (SD)	90.05 (26.62)	89.15 (26.59)	89.78 (26.6)	0.488
BMI, kg/m^2^ (SD)	24.53 (3.83)	24.14 (3.92)	24.41 (3.85)	0.039
Blood pressure, median (SD)				
SBP, mmHg	80.81 (14.08)	78.55 (14.03)	131.4 (21.27)	0.751
DBP, mmHg	132.08 (21.31)	129.79 (21.11)	80.14 (14.10)	0.196
eGFR, ml/min/1.73 m^2^ (SD)	93.61 (53.93)	89.31 (52.96)	92.34 (53.67)	0.011
eGFR < 60 ml/min/1.73 m^2^	3,060 (17.1)	1,686 (22.6)	4,746 (18.7)	<0.001
Moderate and severe renal insufficiency	3,122 (17.4)	1,726 (23.2)	4,848 (19.1)	<0.001
Left atrial diameter (mm) (SD)	40.99 (7.62)	42.50 (8.21)	41.41 (7.82)	<0.001
LVEF (%) (SD)	57.63 (11.14)	56.91 (11.40)	57.42 (11.22)	<0.001
LVEF ≤ 40%, *n* (%)	1,465 (8.2)	629 (8.4)	2,094 (8.3)	0.470
Total cholesterol, mmol/L (SD)	4.51 (1.82)	3.17 (2.17)	3.98 (2.35)	<0.001
LDL-C, mg/dl (SD)	105.84 (28.71)	52.82 (12.40)	90.28 (34.79)	<0.001
CHA_2_DS_2_-VASc score, median (SD)	2.99 (1.74)	3.38 (1.72)	3.01 (1.89)	0.101
HASBLED risk score 0–1	12,440 (69.4)	5,609 (75.3)	18,049 (71.1)	<0.001
HASBLED risk score ≥2	5,491 (30.6)	1,840 (24.7)	7,331 (28.9)	<0.001
AF type, *n* (%)				<0.001
First diagnosed	5,602 (31.2)	2,162 (29.0)	7,764 (30.6)	
Paroxysmal	6,571 (36.6)	2,424 (32.5)	8,995 (35.4)	
Persistent	3,503 (19.5)	1,680 (22.6)	5,183 (20.4)	
Long-standing	2,255 (12.6)	1,183 (15.9)	3,438 (13.5)	
Prior treatment, *n* (%)				
Prior concomitant with antiplatelet	1,172 (6.5)	733 (9.8)	1,905 (7.5)	<0.001
Prior treatment with anticoagulant	3,538 (19.7)	1,791 (24.0)	5,329 (21.0)	<0.001
Prior Antiarrhythmic drugs	1,715 (9.6)	565 (7.6)	2,280 (9.0)	<0.001
Prior Catheter ablation	701 (3.9)	240 (3.2)	941 (3.7)	0.008
Prior cardioversion	113 (0.6)	28 (0.4)	141 (0.6)	0.013
Prior surgical ablation	53 (0.3)	20 (0.3)	73 (0.3)	0.714

AF, atrial fibrillation; CAD, coronary artery disease; PCI, percutaneous coronary intervention; TIA, transient ischemic attack; PAD, peripheral artery disease; COPD, chronic obstructive pulmonary disease; MI, myocardial infarction; BMI, body mass index; DBP, diastolic blood pressure; SBP, systolic blood pressure; eGFR, estimated glomerular filtration rate; LVEF, left ventricular ejection fraction; LDL-C, low-density lipoprotein cholesterol.

### Characteristics of patients stratified by LDL-C level

The mean LDL-C was 90.28 mg/dl in the total population and 52.82 mg/dl and 105.84 mg/dl in the low (<70 mg/dl) and high (≥70 mg/dl) LDL-C groups, respectively. The incidence of bleeding events was 44.6% in the low LDL-C group and 55.4% in the high LDL-C group. The low LDL-C group tended to be older and have a medical history of diabetes mellitus, history of bleeding, liver or kidney disease, higher blood pressure, stroke or TIA, history of major bleeding, and longer hospital stay (all *P* < 0.001), compared with the high LDL-C group. With respect to prior treatment, the low LDL-C group was more likely to have anticoagulant and antiplatelet and less likely to have received antiarrhythmic drugs or to have undergone cardioversion or catheter ablation, compared with the high LDL-C group (all *P* < 0.05). There were no significant between-group differences in ethnicity, tertiary hospitals, medical insurance, history of alcohol use and smoking, blood pressure, LVEF, or prior surgical ablation (all *P* > 0.05).

### Relationship between LDL-C levels and in-hospital bleeding events

The independent predictors of patient outcomes are shown in Table 2. With LDL-C (≥70 mg/dl) as the reference, LDL-C < 70 mg/dl was associated with a higher risk of any bleeding event [adjusted odds ratio [aOR]: 1.63, 95% confidence interval [CI]: 1.12–2.35; *P* = 0.009], major bleeding events (aOR: 1.48, 95% CI: 0.99–2.20; *P* = 0.05), and gastrointestinal bleeding events (aOR: 2.11, 95% CI: 1.27–3.50; *P* = 0.004) in the multivariate logistic regression model (Model 3 in Table 2). These findings persisted in bootstrapping validation (Model 4 in Table 2). No association was observed between LDL-C < 70 mg/dl and intracranial bleeds. Notably, statistical significance was found for ischemic events in patients with LDL-C < 70 mg/dl in all models in Table 2, but not mortality. For the association between anticoagulant history and LDL-C levels, anticoagulants, but not antiplatelets, were associated with an increased probability of bleeding in the low LDL-C group. Additional analyses showed that, other than the original risk factors in the HAS-BLED score, anemia and smoking also increased the risk of bleeding in this group (Table 3). However, a history of cancer and age >65 years did not increase the risk.

**Table 2:** Relationship of low-density lipoprotein cholesterol levels with in-hospital bleeding.

Event	As a continuous variable	*P*-value	As a categorical variable <70 mg/dl with ≥70 mg/dlas reference	*P*-value
Model 1				
Any bleeding	0.99 (0.98,0.99)	0.003	1.91 (1.32, 2.74)	<0.01
Major	0.98 (0.98,0.99)	<0.01	1.72 (1.17, 2.53)	0.005
Intracranial	0.98 (0.97,1.01)	082	0.80 (0.16, 3.97)	0.780
Gastrointestinal	0.98 (0.97,0.99)	<0.01	2,56 (1.57, 4.19)	<.01
All-cause deaths	1.00 (0.99,1.00)	0.274	0.98 (0.62,1.54)	0.946
Ischemic events	1.00 (1.00,1.00)	0.039	0.84 (0.72,0.97)	0.023
Clinical outcome	1.00 (0.99,1.00)	0.324	0.94 (0.82,1.08)	0.438
Model 2				
Any bleeding	0.99 (0.98,0.99)	0.02	1.03 (1.01,1.05)	<0.01
Major	0.99 (0.98,0.99)	0.002	1.55 (1.05,2.28)	0.02
Intracranial	1.00 (0.98,1.02)	0.93	0.65 (0.13,3.28)	0.60
Gastrointestinal	0.98 (0.97,0.99)	<0.01	1.06 (1.03,1.08)	<0.01
All-cause deaths	1.00 (0.99,1.00)	0.91	0.80 (0.51,1.25)	0.333
Ischemic events	1.00 (1.00,1.00)	0.001	0.77 (0.66,0.89)	<0.01
Clinical outcome	1.00 (1.00,1.00)	0.015	0.86 (0.75,0.99)	0.037
Model 3				
Any bleeding	0.99 (0.98,1.00)	0.08	1.63 (1.12,2.35)	<0.01
Major	0.99 (0.98,0.99)	0.005	1.48 (0.99,2.20)	0.05
Intracranial	0.99 (0.97,1.01)	0.775	0.80 (0.15,4.11)	0.780
Gastrointestinal	0.98 (0.97,0.99)	0.001	2.11 (1.27,3.50)	0.004
All-cause deaths	1.00 (0.99,1.00)	0.848	0.78 (0.49,1.25)	0.31
Ischemic events	1.00 (1.00,1.00)	0.001	0.72 (0.62,0.84)	<0.01
Clinical outcome	1.00 (1.00,1.00)	0.003	0.81 (0.70,0.93)	0.003
Model 4				
Any bleeding	0.99 (0.98,1.00)	0.07	1.63 (1.13,2.36)	0.009
Major	0.99 (0.98,0.99)	0.005	1.49 (1.00,2.21)	0.046
Intracranial	0.99 (0.97,1.01)	0.768	0.81 (0.15,4.17)	0.801
Gastrointestinal	0.98 (0.97,0.99)	0.001	2.12 (1.28,3.52)	0.003
All-cause deaths	1.00 (0.99,1.00)	0.80	0.77 (0.48,1.23)	0.28
Ischemic events	1.00 (1.00,1.00)	0.001	0.73 (0.63,0.85)	<0.01
Clinical outcome	1.00(1.00,1.00)	0.006	0.81(0.71,0.94)	0.005

Model 1: crude model.

Model 2: adjusted for age and sex.

Model 3: adjusted for sex, age, tertiary hospital, hospital stay, smoking history, drinking history, medical history including Diabetes mellitus, hypertension, stroke or TIA, heart failure, COPD, chronic liver disease, bleeding history, chronic kidney disease, anemia, cancer, LVEF ≤ 40%, prior antiarrhythmic drugs, prior catheter ablation, prior surgical ablation, prior statins, prior antiplatelet, prior anticoagulant.

Model 4: adjusted for covariates in model 3 with 1,000 bootstrapping replications performed.

AF, atrial fibrillation; TIA, transient ischemic attack; COPD, chronic obstructive pulmonary disease; LVEF, left ventricular ejection fraction; LDL-C, low-density lipoprotein cholesterol.

**Table 3:** Univariable analysis of factors associated with in-hospital bleeding in AF patients with LDL-C < 70 mg/dl.

Variables	OR(95%CI)	*P*-value	P-interaction
Age >65	1.36 (0.61–3.03)	0.18	0.05
Smoke	2.31 (1.16–4.58)	0.01	0.83
Drinking	0.86 (0.31–2.41)	0.78	0.40
Cancer	0.38 (0.11–1.24)	0.10	0.20
Anemia	0.15 (0.06–0.39)	<0.01	<0.01
Cr >200	4.95 (2.31–10.59)	<0.01	0.97
Anticoagulant history	0.53 (0.30–0.93)	0.02	0.57
Antiplatelets	0.73 (0.32–1.62)	0.44	0.82
Bleeding history	15.48 (7.90–30.07)	<0.01	0.15
DM	1.01 (0.51–1.96)	0.96	0.29
Hypertension	0.75 (0.44–1.28)	0.29	0.29
Tertiary hospitals	1.63 (0.98–2.71)	0.05	0.47

AF, atrial fibrillation; DM, diabetes mellitus.

**Figure 2:**
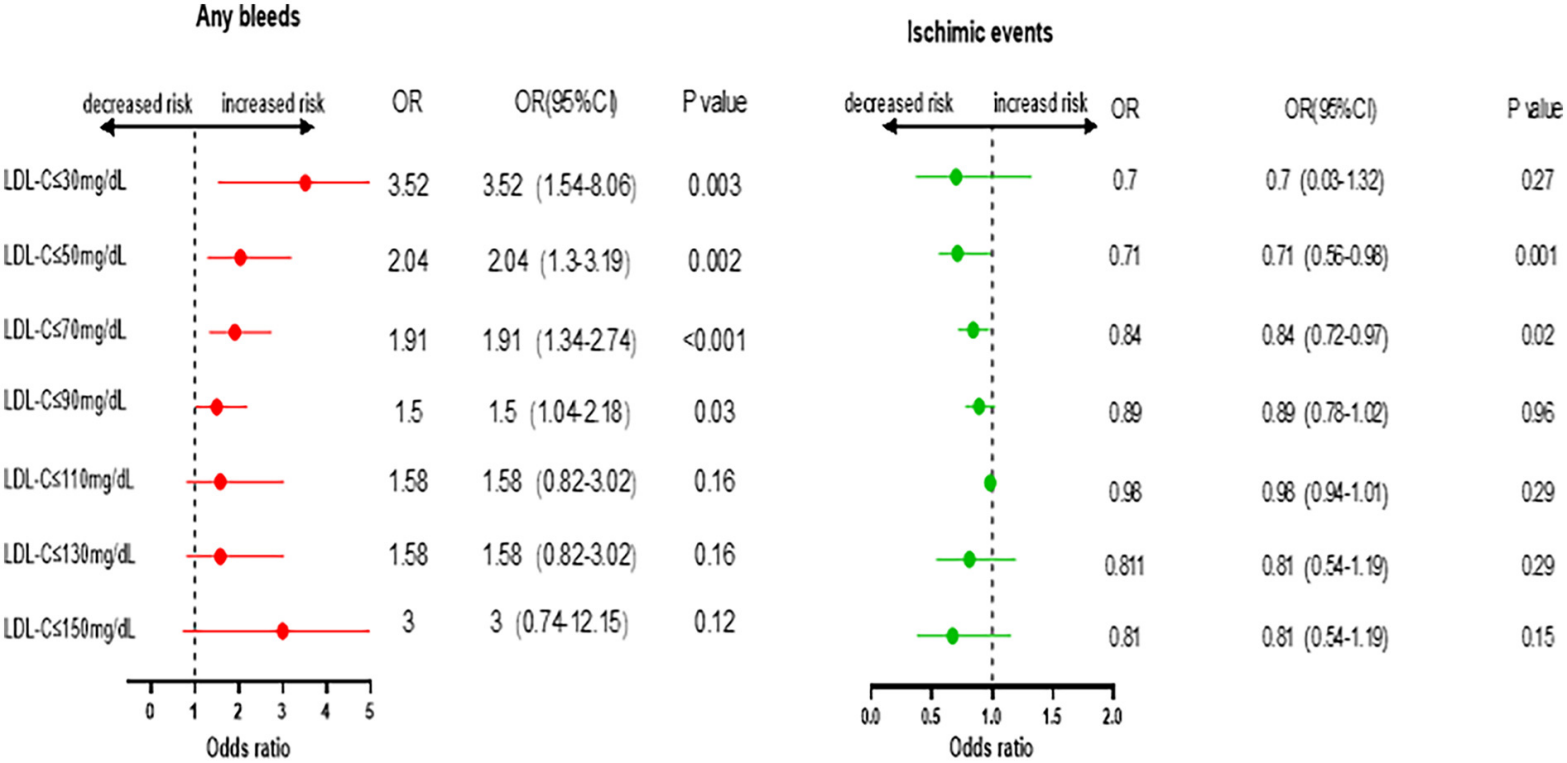
Odds ratio and 95% confidence intervals for Any bleeds and ischemic events. Comparing lower than the endpoint with higher as reference.

The incidence rates of in-hospital bleeding and ischemic events significantly differed by LDL-C level category (both *P* < 0.05). After logistic regression, the level of LDL-C (70 mg/dl) and lower increased the risk of bleeding events with the lowest risk of 110–130 mg/dl as a reference, and the level of LDL-C and higher increased the risk of ischemic events with the lowest risk of 30–50 mg/dl as a reference (Figure 1). For further study, when comparing each endpoint among the LDL-C level categories (lower than the endpoint with higher as a reference), the results showed a constant increasing trend of risk for bleeding at LDL-C levels <70 mg/dl. This effect stopped being significant when the LDL-C levels reached ≥70 mg/dl (*P* > 0.05). Meanwhile, an opposite trend was found for ischemic events, with a decreasing trend of risk at LDL-C levels <70 mg/dl, and this stopped being significant when the LDL-C levels reached ≥70 mg/dl (Figure 2).

### Nonlinear relationship between LDL-C levels and study outcomes

**Figure 3:**
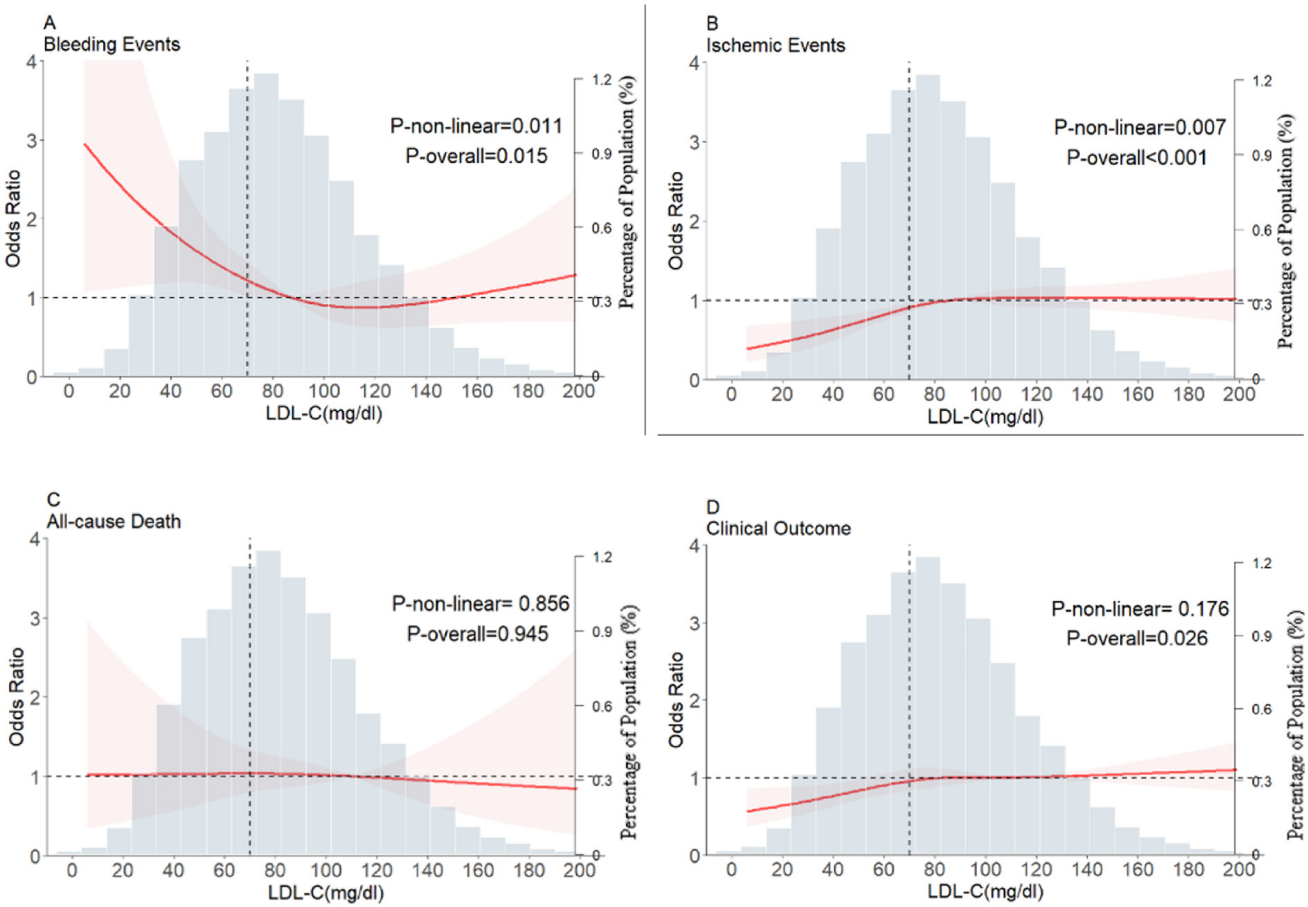
Restricted cubic spline plots for major bleeding events, ischemic events, all-cause death, and clinical outcome according to LDL-C levels after covariate adjustment. **(A)** Bleeding events, **(B)** ischemic events, **(C)** all-cause death, **(D)** clinical outcomes. The background histograms (light grey color) represent the percentage of the density distribution of LDL-C in the study population (right *y*-axis). The heavy red lines represent the estimated adjusted odds ratios, with the pink-shaded ribbons denoting the 95% confidence intervals. The vertical dotted lines indicate the threshold value for LDL-C, at 70 mg/dl. The horizontal dotted lines represent the OR of 1.0. The reference point for the lowest risk is 110 mg/dl (Knot = 4). OR, Odds ratio; LDL-C, low-density lipoprotein cholesterol.

**Figure 4:**
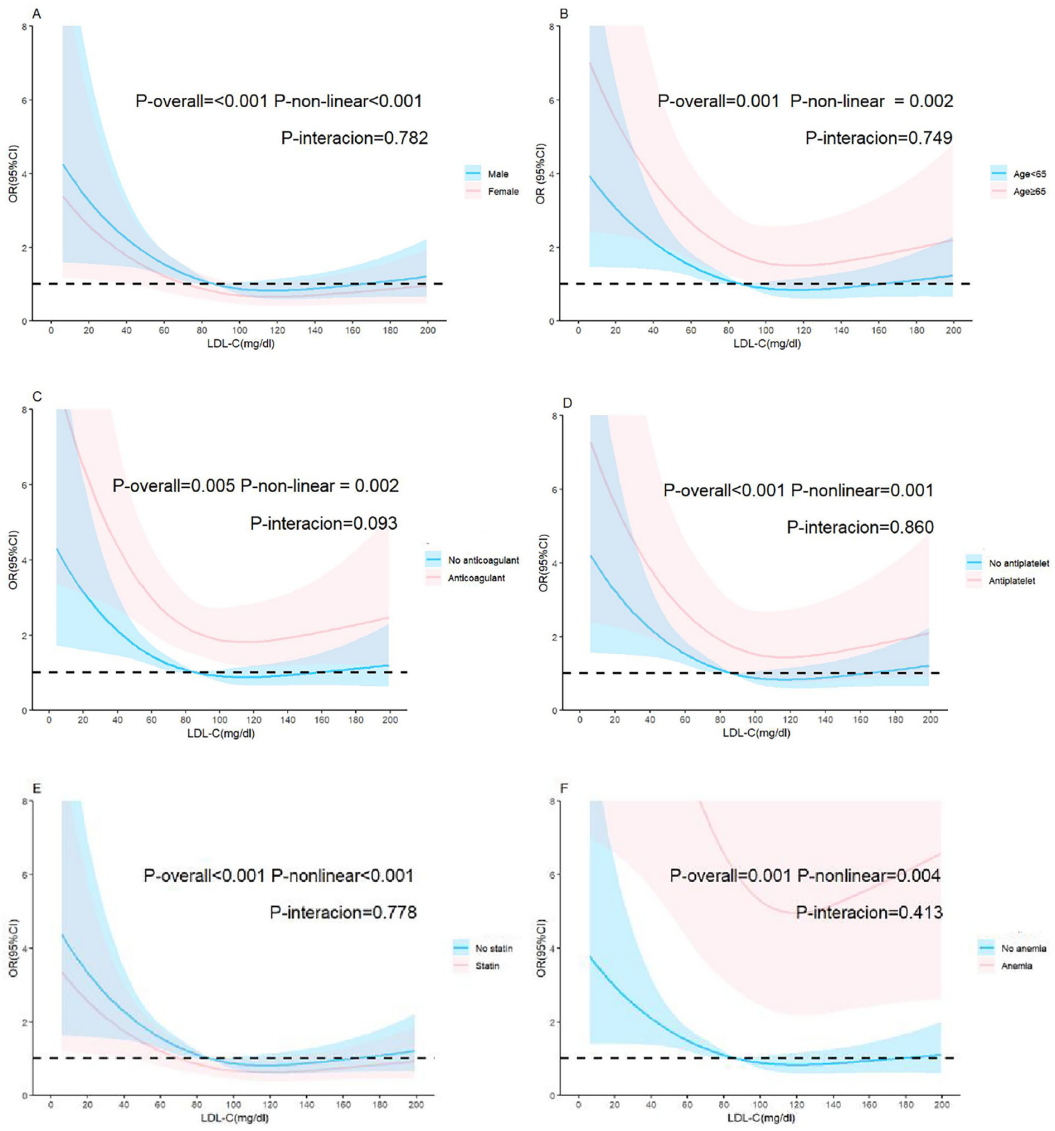
Adjusted relationship of LDL-C and bleeding in the restricted cubic spline subgroups. **(A)** Sex, **(B)** age, **(C)** anticoagulant intake, **(D)** antiplatelet intake, **(E)** statin intake, **(F)** anemia. The reference value (OR = 1) is set as the median value for each subgroup. The solid lines represent the adjusted ORs of LDL-C levels across the entire range, and the shaded areas represent the corresponding 95% confidence intervals. OR, Odds ratio; LDL-C, low-density lipoprotein cholesterol.

The reference point was set at the lowest risk of bleeding in each plot (LDL-C level of 110 mg/dl). LDL-C levels were found to have a nonlinear, L-shaped relationship with in-hospital bleeding, with a higher risk at lower LDL-C levels (P for nonlinearity = 0.011, P for overall = 0.015). Further, there was a nonlinear relationship between LDL-C levels and ischemic events, with lower risk at lower LDL-C levels (P for nonlinearity = 0.007, P for overall < 0.001). However, LDL-C levels did not demonstrate a nonlinear relationship with all-cause death and clinical outcomes (P for nonlinearity = 0.856 and =0.176, respectively) (Figure 3). The nonlinear relationship between LDL-C levels and the risk of bleeding outcomes remained in the subgroups stratified by sex, age, anticoagulant use, antiplatelet use, statin use, anemia (Figure 4) and Diabetes mellitus, hypertension and eGFR < 60 (Supplementary Figure S1). We also conducted subgroup analyses stratified by HAS-BLED scores. The results showed no significant interaction between LDL-C levels and HAS-BLED categories (P-interaction = 0.158), while the nonlinear relationship remained significant (P-overall = 0.002; P-nonlinear = 0.004) (Supplementary Figure S1).

## Discussion

The principal findings of this large-scale national AF registry-based dataset were as follows. First, 0.45% of the patients with AF had bleeding, and this rate was markedly lower than that in Western cohorts. Second, after adjusting for covariates, with LDL-C levels ≥70 mg/dl as the reference, LDL-C levels <70 mg/dl were associated with a higher risk of bleeding and a lower risk of ischemic events. Low LDL-C levels were strongly associated with the risk of gastrointestinal bleeding but not with that of intracranial bleeding. Third, anticoagulants, anemia, and smoking, in addition to the original risk factors in the HAS-BLED score, were associated with increased probabilities of bleeding in patients with lower LDL-C levels but not in those with higher LDL-C levels. Fourth, the restricted cubic spline analyses found an L-shaped relationship between LDL-C levels and in-hospital bleeding. The nonlinear relationship between LDL-C levels and the risk of bleeding outcomes persisted among the subgroups. These findings have important implications for the current management of AF.

Low LDL-C levels are associated with clinical outcomes, and some studies have attempted to determine what levels are too low (16). Recent evidence indicates that low LDL-C levels are associated with poor bleeding outcomes (8–10, 17). With the prevalence of statin use in patients with AF and cardiovascular disease (CVD), marked lowering of LDL-C levels has become realistic. In contrast to previous findings, our research suggests that low LDL-C levels are related to bleeding events in patients with AF. Although the mechanisms by which low LDL-C can promote bleeding are still unclear, some possible explanations exist. First, low LDL-C levels are strongly related to more microbleeds and erythrocyte fragility due to low cholesterol in the erythrocyte membrane.Disrupting intracellular cholesterol hemostasis might result in defect of terminal erythroid differentiation *in vivo* (18, 19). Second, LDL-C, a vital part of the platelet cell membrane, plays an essential role in platelet activation, tissue factor expression, and impaired coagulation function (20, 21).Third, low LDL-C levels are metabolically associated with Proprotein convertase subtilisin/kexin type 9 (PCSK9) concentrations (21–23). PCSK9 boosts platelet activation by binding with platelet CD36. Consequently, when LDL-C levels are low, and plasma PCSK9 levels decrease, the PCSK9/CD36-mediated downstream signaling pathways of cyclooxygenase-1/thromboxane A2 are inhibited. This phenomenon leads to increased platelet inhibition and impaired blood clotting mechanisms (23).

We found that among all kinds of bleeding events (intracranial, gastrointestinal, and others), gastrointestinal bleeding events were more directly related to low LDL-C levels than were intracranial bleeding events. These findings differ from those of some studies (9, 24), and this can be explained as follows: First, only a small number of intracranial hemorrhages were recorded in our trial. Second, the patients recruited for the CCC-AF project were mainly from the cardiology department, whereas patients with ischemic or hemorrhagic stroke as their first diagnosis without knowledge of AF in the neurology department were not included.

We found that in patients with low LDL-C levels (<70 mg/dl), anemia, in addition to the risk factors in the HAS-BLED score, also increased the risk of bleeding. The *P*-value for interaction between LDL-C < 70 mg/dl and anemia was <0.01. Low hemoglobin levels were found in some research to be a predictor for all-cause death and composite events such as increased hospitalization, bleeding, and thromboembolic events, as reported in previous studies (25, 26). In our sensitivity analysis adjusted for anemia, the strength of the relationship between low LDL-C and major bleeding events was unchanged. Notably, patients with LDL-C levels <70 mg/dl and a history of cancer did not have an increased risk of bleeding. However, some previous studies concluded that cancer complicates the clinical history of patients with AF and increases the risks of all-cause mortality, major bleeding, and intracranial hemorrhage in these patients. The risk of bleeding exceeds that of thromboembolism (27, 28).

The restricted cubic spline model showed a nonlinear and L-shaped relationship between LDL-C levels and bleeding events. Consistent with the theory that low LDL-C levels are an indirect marker of certain body degeneration traits, the relationship between low LDL-C levels and the risk of bleeding was more evident in the anemia and age >65 years subgroups. The frailty index has been studied and recommended as a tool to predict unplanned hospitalizations and other adverse outcomes, such as all-cause mortality and bleeding, but not stroke (29). Hemoglobin levels are also an independent predictor of all-cause death and composite events, such as thromboembolism and major hemorrhage (25, 26). To exclude interaction effect between covariates and LDL-C, we did subgroups including anticoagulant use, antiplatelet use, statin use, and anemia. The interaction *P*-value showed none. Of note, subgroup analyses stratified by HAS-BLED score demonstrated that a score ≥2 was associated with a higher bleeding risk, further validating HAS-BLED as a reliable tool for bleeding risk assessment. Additionally, the interaction *P*-value suggested that LDL-C may be an independent risk factor for bleeding.

We conducted a sensitivity analysis across the full range of LDL-C levels to identify the LDL-C levels associated with a higher risk of bleeding events. By comparing each endpoint of the LDL-C groups (lower than the endpoint with higher as a reference), we found an increasing trend for bleeding with a steady increase in risk at LDL-C levels below 70 mg/dl. This effect stopped being significant when LDL-C levels reached ≥70 mg/dl. In contrast, an opposite trend was found for ischemic events, with a consistent decrease in risk at LDL-C levels <70 mg/dl, and this stopped being significant when the levels reached ≥70 mg/dl. These results show that there is no safe zone for bleeding and ischemic events. Maintaining LDL-C levels >70 mg/dl may be best to prevent bleeding. However, once patients are assessed to have a high risk of ASCVD, LDL-C levels should still be strictly monitored to be ≤70 mg/dl because the incidence of bleeding is lower than that of ASCVD.

With the wider application of PCSK9 inhibitors, there will be a high prevalence of patients with LDL-C levels ≤70 mg/dl. It is essential to provide physicians with guidelines to identify patients requiring careful monitoring of their LDL-C levels and the appropriate range during clinical follow-up.

### Strengths and limitations

The major strengths of this study are its large sample size and coverage, which provided sufficient power to explain the relationship between LDL-C levels and in-hospital bleeding events and enabled the results to be representative of the national population. Nevertheless, we acknowledge some limitations. First, this study has inherent limitations associated with an observational design, including the inability to establish a causal association between low LDL-C levels and bleeding risk in AF. Despite our efforts to adjust for many important confounders, there is potential for residential and unmeasured confounding. Second, although many studies found that low LDL-C levels were related to bleeding, more evidence of a clear biological mechanism by which low LDL-C can promote bleeding is warranted. Third, this study only included in-hospital events and lacked long-term follow-up, therefore, the bleeding events and mortality rate were low. Thus, our study may be underpowered to fully evaluate the association between low LDL-C levels and bleeding events.

## Conclusion

Our results demonstrate an L-shaped relationship between LDL-C levels and bleeding events, with lower LDL-C levels associated with a higher risk of bleeding events. LDL-C levels may need to be considered in the risk stratification and management of patients with AF.

## Data Availability

The data analyzed in this study is subject to the following licenses/restrictions: raw data are available upon reasonable request with the corresponding author. Requests to access these datasets should be directed to De-yong Long: dragon2008@vip.sina.com.
