# Supplementary material for: Low-density lipoprotein cholesterol levels and in-hospital bleeding in patients with atrial fibrillation: findings from CCC-AF project
Source: Front Cardiovasc Med. 2025 Jun 12;12:1574796. doi: 10.3389/fcvm.2025.1574796 (PMC12199265; doi:10.3389/fcvm.2025.1574796)
Supplement: Supplementary file 1 [file Datasheet1.pdf]

Supplementary Material

Low-density lipoprotein cholesterol levels and in-hospital bleeding in patients with atrial fibrillation: Findings from CCC-AF project

Supplementary Figure 1 Adjusted relationship of LDL-C and bleeding in the restricted cubic spline subgroups stratified by HAS-BLED score.

Supplementary Table 1. Investigators of the Improving Care for Cardiovascular Disease in China-Atrial Fibrillation (CCC-AF) project.

Supplementary Figure 1 Adjusted relationship of LDL-C and bleeding in the restricted cubic spline subgroups.

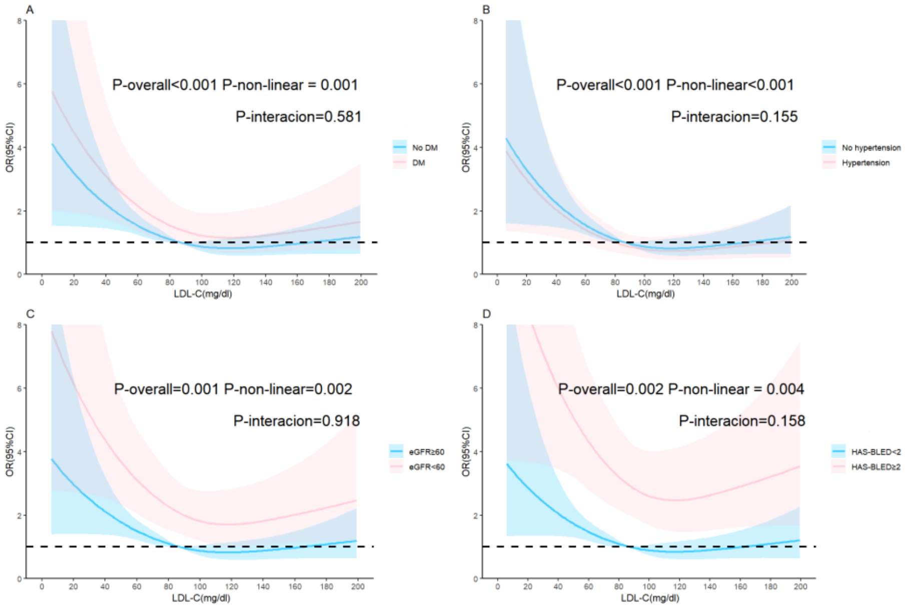

Supplementary Table 1. Investigators of the Improving Care for Cardiovascular Disease in China-Atrial Fibrillation (CCC-AF) project.

ID	Hospitals	Territories	Provinces	City	Investigator
1	Peking University First Hospital	Northern China	Beijing	Beijing	Jie Jiang
2	Beijing Anzhen Hospital, Capital Medical University	Northern China	Beijing	Beijing	Shaoping Nie, Xiaohui Liu
3	The First Affiliated Hospital of Bengbu Medical College	Eastern China	Anhui	Bengbu	Honhju Wang
4	Beijing Friendship Hospital, Capital Medical University	Northern China	Beijing	Beijing	Hongwei Li
5	The First Affiliated Hospital of Chongqing Medical University	Southwest China	Chongqing	Chongqing	Suxin Luo
6	Changhai Hospital of Shanghai	Eastern China	Shanghai	Shanghai	Xianxian Zhao
7	Xinqiao Hospital, Third Military Medical University	Southwest China	Chongqing	Chongqing	Bin Cui, Lan Huang
8	Dongguan People's Hospital	Southern China	Guangdong	Dongguan	Jianfeng Ye
9	Zhongda Hospital, Southeast University	Eastern China	Jiangsu	Nanjing	Genshan Ma
10	Gansu Provincial Hospital	Northwest China	Gansu	Lanzhou	Ping Xie
11	Guangdong General Hospital	Southern China	Guangdong	Guangzhou	Jiyan Chen
12	The First Affiliated Hospital of Guangxi Medical University	Southern China	Guangxi	Nanning	Lang Li
13	The People's Hospital of Guangxi Zhuang Autonomous Region	Southern China	Guangxi	Nanning	Yingzhong Lin
14	Panyu Hospital of Chinese Medicine	Southern China	Guangdong	Guangzhou	Jianhao Li
15	The Affiliated Hospital of Guizhou Medical University	Southwest China	Guizhou	Guiyang	Lirong Wu
16	The 2nd Affiliated Hosiptal of Harbin Medical University	Northeast China	Heilongjiang	Harbin	Bo Yu
17	Navy General Hospital	Northern China	Beijing	Beijing	Tianchang Li
18	Haikou People's Hospital	Southern China	Hainan	Haikou	Moshui Chen
19	Hainan General Hospital	Southern China	Hainan	Haikou	Bin Li

20	The First Hospital of Handan	Northern China	Hebei	Handan	Shuanli Xin
21	Hebei General Hospital	Northern China	Hebei	Shijiazhuang	Xiaoyong Qi
22	The Second Hospital of Hebei Medical University	Northern China	Hebei	Shijiazhuang	Xianghua Fu
23	The First Affiliated Hospital of Henan University of Science and Technology	Central China	Henan	Luoyang	Pingshuan Dong
24	Henan Provincial People's Hospital	Central China	Henan	Zhengzhou	Chuanyu Gao
25	Chenzhou First People's Hospital	Central China	Hunan	Chenzhou	Qiaoqing Zhong
26	Hunan Provincial People's Hospital	Central China	Hunan	Changsha	Ying Guo
27	West China Hospital of Sichuan University	Northwest China	Sichuan	Chengdu	Xiaoping Chen
28	Huai'an First People's Hospital	Eastern China	Jiangsu	Huai'an	Shuren Ma
29	The First Hospital of Jilin University	Northeast China	Jilin	Changchun	Yang Zheng
30	The Second Hospital of Jilin University	Northeast China	Jilin	Changchun	Bin Liu
31	Nanjing Drum Tower Hospital, The Affiliated Hospital of Nanjing University Medical School	Eastern China	Jiangsu	Nanjing	Biao Xu, Guangshu Han
32	Jiangsu Province Hospital	Eastern China	Jiangsu	Nanjing	Zhijian Yang
33	First Affiliated Hospital of the People's Liberation Army General Hospital	Northern China	Beijing	Beijing	Miao Tian
34	The First Affiliated Hospital of Lanzhou University	Northwest China	Gansu	Lanzhou	Zheng Zhang
35	The First Affiliated Hospital of Liaoning Medical University	Northeast China	Liaoning	Jinzhou	Guizhou Tao
36	China Meitan General Hospital	Northern China	Beijing	Beijing	Di Wu
37	The First Affiliated Hospital to Nanchang University	Eastern China	Jiangxi	Nanchang	Zeqi Zheng
38	The Second Affiliated Hospital to Nanchang University	Eastern China	Jiangxi	Nanchang	Xiaoshu Cheng

39	Nanfang Hospital of Southern Medical University	Southern China	Guangdong	Guangzhou	Yuqing Hou
40	Inner Mongolia People's Hospital	Northern China	Inner Mongolia	Hohhot	Xingsheng Zhao
41	People’s Hospital of Qinghai Province	Northwest China	Qinghai	Xining	Rong Chang
42	Binzou City Center Hospital	Eastern China	Shandong	Binzhou	Lijun Meng
43	Shanxi Provincial People's Hospital	Northern China	Shanxi	Taiyuan	Chunlin Lai
44	Shanxi Cardiovascular Hospital	Northern China	Shanxi	Taiyuan	Bao Li
45	The Second Hospital of Shanxi Medical University	Northern China	Shanxi	Taiyuan	Zhiming Yang
46	The Ninth Hospital Affiliated to Shanghai Jiaotong University School of Medicine	Eastern China	Shanghai	Shanghai	Changqian Wang
47	Shanghai Sixth People's Hospital	Eastern China	Shanghai	Shanghai	Shixin Ma
48	Tongren Hospital Affiliated to Shanghai Jiaotong University School of Medicine	Eastern China	Shanghai	Shanghai	Li Jiang
49	The General Hospital of Shenyang Military Region	Northeast China	Liaoning	Shenyang	Yaling Han
50	The Third Hospital of Shijiazhuang	Northern China	Hebei	Shijiazhuang	Zhenguo Ji
51	North Jiangsu People's Hospital	Eastern China	Jiangsu	Yangzhou	Shenghu He
52	General Hospital of TISCO	Northern China	Shanxi	Taiyuan	Huifeng Wang
53	Tianjin Chest Hospital	Northern China	Tianjin	Tianjin	Yin Liu
54	Teda International Cardiovascular Hospital	Northern China	Tianjin	Tianjin	Wenhua Lin
55	Tianjin Medical University General Hospital	Northern China	Tianjin	Tianjin	Yuemin Sun
56	Wuxi People's Hospital	Eastern China	Jiangsu	Wuxi	Zhenyu Yang
57	The First Affiliated Hospital of Xi’an Jiaotong University	Northwest China	Shaanxi	Xi'an	Zuyi Yuan
58	Xijing Hospital	Northwest China	Shaanxi	Xi'an	Ling Tao

59	Southwest Hospital, Third Military Medical University	Southwest China	Chongqing	Chongqing	Zhiyuan Song
60	Hospital of Xinjiang Production & Construction Corps	Northwest China	Xinjiang	Urumchi	Junming Liu
61	The First Teaching Hospital of Xinjiang Medical University	Northwest China	Xinjiang	Urumchi	Yitong Ma
62	Xinjiang Uygur Autonomous Region People's Hospital	Northwest China	Xinjiang	Urumchi	Guoqing Li
63	The Affiliated Hospital of Xuzhou Medical College	Eastern China	Jiangsu	Xuzhou	Zhirong Wang
64	People's Hospital of Yuxi City	Southwest China	Yunnan	Yuxi	Yinglu Hao
65	The Second People's Hospital of Yunnan Province	Southwest China	Yunnan	Kunming	Minghua Han
66	Sir Run Run Shaw Hospital, College of Medicine, Zhejiang University	Eastern China	Zhejiang	Hangzhou	Guosheng Fu
67	The Second Affiliated Hospital of Zhengzhou University	Central China	Henan	Zhengzhou	Yulan Zhao
68	The First Affiliated Hospital of Zhengzhou University	Central China	Henan	Zhengzhou	Ling Li
69	The Third Xiangya Hospital of Central South University	Central China	Hunan	Changsha	Weihong Jiang
70	Sun Yat-sen Memorial Hospital, Sun Yat-sen University	Southern China	Guangdong	Guangzhou	Jingfeng Wang
71	Baogang Hospital	Northern China	Inner Mongolia	Baotou	Yongdong Li
72	Zhejiang Provincial Hospital of TCM	Eastern China	Zhejiang	Hangzhou	Wei Mao
73	Affiliated Hospital of Qinghai University	Northwest China	Qinghai	Xining	Weijun Liu
74	Anhui Provincial Hospital	Eastern China	Anhui	Hefei	Likun Ma
75	Anyang District Hospital	Central China	Henan	Anyang	Hui Liu
76	The Third the People's Hospital of Bengbu	Eastern China	Anhui	Bengbu	Gengsheng Sang
77	Cangzhou Central Hospital	Northern China	Hebei	Cangzhou	Zesheng Xu

78	The First People's Hospital of Changde	Central China	Hunan	Chang de	Yi Huang
79	Dalian Municipal Central Hospital	Northeast China	Liaonin g	Dalian	Hailong Lin
80	The Second hospital of Dalian Medical University	Northeast China	Liaonin g	Dalian	Peng Qu
81	The First Affiliated hospital of Dalian Medical University	Northeast China	Liaonin g	Dalian	Yanzong Yang
82	Longyan First Hospital	Eastern China	Fujian	Longy an	Kaihong Chen
83	The First Affiliated Hospital of Fujian Medical University	Eastern China	Fujian	Fuzho u	Jinzi Su
84	Affiliated Hospital of Guangdong Medical College	Southern China	Guangd ong	Guang zhou	Keng Wu
85	The First Affiliated Hospital of Guangzhou Medical College	Southern China	Guangd ong	Guang zhou	Wei Wang
86	The Third Affiliated Hospital of Guangzhou Medical College	Southern China	Guangd ong	Guang zhou	Ximing Chen
87	Guizhou Provincial People's Hospital	Northwest China	Guizhou	Guiyan g	Qiang Wu
88	The Central Hospital of Zhoukou	Central China	Henan	Zhouk ou	Hualing Liu
89	The Central Hospital of Jilin	Northeast China	Jilin	Chang chun	Shuangbin Li
90	The First People's Hospital of Jining	Eastern China	Shandon g	Jining	Xiaofei Sun
91	Affiliated Hospital of Jiangsu University	Eastern China	Jiangsu	Zhenji ang	Jinchuan Yan
92	Jiangxi Provincial People's Hospital	Eastern China	Jiangxi	Nanch ang	Lang Ji
93	The People's Hospital of Liaoning Province	Northeast China	Liaonin g	Shenya ng	Zhanquan Li
94	The First Affiliated Hospital of Liaoning University of Traditional Chinese Medicine	Northeast China	Liaonin g	Shenya ng	Ping Hou
95	Liaocheng People's Hospital	Eastern China	Shandon g	Liaoch eng	Chunyan Zhang
96	Linyi People's Hospital	Eastern China	Shandon g	Linyi	Zhihong Ou
97	Mudanjiang Cardiovascular Disease Hospital	Northeast China	Heilongj iang	Mudan jiang	Jianwen Liu

98	The First People's Hospital of Nanning City	Southern China	Guangxi	Nanning	Jinru Wei
99	Ningxia People's Hospital	Northwest China	Ningxia	Yinchuan	Hong Luan
100	Qingdao Municipal Hospital	Eastern China	Shandong	Qingdao	Jun Guan
101	Quanzhou First Hospital	Eastern China	Fujian	Quanzhou	Rong Lin
102	The First Affiliated Hospital of Xiamen University	Eastern China	Fujian	Xiamen	Qiang Xie
103	Xiamen Cardiovascular Disease Hospital	Eastern China	Fujian	Xiamen	Yan Wang
104	Qilu Hospital of Shandong University	Eastern China	Shandong	Jinan	Jifu Li
105	Yantaishan hospital	Eastern China	Shandong	Yantai	Juexin Fan
106	Zhongshan Hospital Affiliated to Fudan University	Eastern China	Shanghai	Shanghai	Junbo Ge
107	Shanghai East Hospital Affiliated to Tongji University	Eastern China	Shanghai	Shanghai	Xuebo Liu
108	The Central Hospital of Shaoyang	Central China	Hunan	Shaoyang	Zewei Ouyang
109	Central Hospital Affiliated to Shenyang Medical College	Northeast China	Liaoning	Shenyang	Man Zhang, Kaiming Chen
110	The First Affiliated Hospital of Soochow University	Eastern China	Jiangsu	Suzhou	Xiangjun Yang
111	The Second Affiliated Hospital of Soochow University	Eastern China	Jiangsu	Suzhou	Weiting Xu
112	The Central Hospital of Taiyuan	Northern China	Shanxi	Taiyuan	Xiaoping Chen
113	Tangshan Gongren Hospital	Northern China	Hebei	Tangshan	Zheng Ji
114	The First Affiliated Hospital of Wannan Medical College	Eastern China	Anhui	Wuhu	Xingsheng Tang
115	The First Affiliated Hospital of Wenzhou Medical University	Eastern China	Zhejiang	Wenzhou	Weijian Huang
116	Wuzhou People's Hospital	Southern China	Guangxi	Wuzhou	Shaowu Ye
117	Renmin Hospital of Wuhan University	Central China	Hubei	Wuhan	Hong Jiang
118	Xiangtan City Central Hospital	Central China	Hunan	Xiangtan	Lilong Tang

11<br>9	The Central Hospital of Xuzhou	Eastern China	Jiangsu	Xuzho<br>u	Peiying Zhang
12<br>0	Affiliated Hospital of Yan'an University	Northwest China	Shaanxi	Yan'an	Xiaochuan Ma
12<br>1	Yancheng Third People's Hospital	Eastern China	Jiangsu	Yanch<br>eng	Chunyang Wu
12<br>2	Yichang Central Hospital	Central China	Hubei	Yichan<br>g	Jiawang Ding
12<br>3	The First People's Hospital of Yunnan Province (Kunhua Hospital)	Northwest China	Yunnan	Kunmi<br>ng	Hong Zhang
12<br>4	Hospital 463 of Chinese People's Liberation Army	Northeast China	Liaonin<br>g	Shenya<br>ng	Bosong Yang
12<br>5	The First Affiliated Hospital of China Medical University	Northeast China	Liaonin<br>g	Shenya<br>ng	Yingxian Sun
12<br>6	The Fourth Affiliated Hospital of China Medical University	Northeast China	Liaonin<br>g	Shenya<br>ng	Yuanzhe Jin
12<br>7	The Second Xiangya Hospital of Central South University	Central China	Hunan	Chang<br>sha	Daoquan Peng
12<br>8	Xiangya Hospital Central South University	Central China	Hunan	Chang<br>sha	Tianlun Yang
12<br>9	Zhoushan People's Hospital	Eastern China	Zhejian<br>g	Zhous<br>han	Guoxiong Chen
13<br>0	Chengdu Third People’s Hospital	Northwest China	Sichuan	Cheng<br>du	Jiong Tang
13<br>1	Tangdu Hospital of The Fourth Military Medical University	Northwest China	Shaanxi	Xi'an	Xue Li
13<br>2	The First Hospital of Haerbin City	Northeast China	Heilongj<br>iang	Harbin	Lin Wei
13<br>3	The First Affiliated Hospital of Jiamusi University	Northeast China	Heilongj<br>iang	Jiamus<br>i	Zhaofa He
13<br>4	The Central Hospital of Panzhihua	Northwest China	Sichuan	Panzhi<br>hua	Dawen Xu
13<br>5	Wuhan Asia Heart Hospital	Central China	Hubei	Wuhan	Xi Su
13<br>6	Sichuan Provincial People’s Hospital	Northwest China	Sichuan	Cheng<br>du	Jianhong Tao
13<br>7	The Central Hospital of Mianyang	Northwest China	Sichuan	Miany<br>ang	Caidong Luo
13<br>8	The First Hospital of Jiamusi	Northeast China	Heilongj<br>iang	Jiamus<br>i	Guixia Zhang

139	Beijing Tsinghua Changgung Hospital	Northern China	Beijing	Beijing	Ping Zhang
140	Chongqing Hechuan District People's Hospital	Southwest China	Chongqing	Chongqing	Xin Tang
141	Yuzhou City Central Hospital	Central China	Henan	Xuchang	Qinfeng Su
142	Jianshui County People's Hospital	Southwest China	Yunnan	Honghe	Weiqing Fan
143	Dunhua City Hospital	Northeast China	Jilin	Yanbian	Fanju Meng
144	Shenyang City Electricity Central Hospital	Northeast China	Liaoning	Shenyang	Jing Xu
145	Shanghai Jingan District Shibei Hospital	Eastern China	Shanghai	Shanghai	Bin Wang
146	Beijing Fangshan District First Hospital	Northern China	Beijing	Beijing	Xuemei Peng
147	Hebei Daming County People's Hospital	Northern China	Hebei	Handan	Haiping Guo
148	Jiangsu Binhai County People's Hospital	Eastern China	jiangsu	Yancheng	Yonglin Zhang
149	The First People's Hospital of Longquanyi District	Southwest China	Sichuan	Chengdu	Wei Tuo
150	Guangxi Hengxian County People's Hospital	Southern China	Guangxi	Nanning	Xianan Zhang
151	Hunan Changsha County First People's Hospital	Central China	Hunan	Changsha	Siding Wang
152	People's Hospital of Wugang	Central China	Hunan	Shaoyang	JiaoMei Yang
153	Longhui County People's Hospital	Central China	Hunan	Shaoyang	Xiaojun Wang
154	Heilongjiang Fujin City Central Hospital	Northeast China	Heilongjiang	Jiamusi	Jiyan Yin
155	Dalian Fourth People's Hospital	Northeast China	Liaoning	Dalian	Huifang Zhang
156	General Hospital of Guangzhou Military Command	Southern China	Guangdong	Guangzhou	Yanlie Zheng
157	The First People's Hospital of Horqin District, Tongliao City	Northern China	Inner Mongolia	Tongliao	Junping Fang
158	Guiyang Sixth People's Hospital	Southwest China	Guizhou	Guiyang	Kalan Luo

159	Geological Mining Hospital of Hunan Province	Central China	Hunan	Changsha	Naiyi Liang
160	Zhangzhou Municipal Hospital of Fujian Province	Eastern China	Fujian	Zhangzhou	Changyong Liu
161	Jining City Yanzhou District People’s Hospital	Eastern China	Shandong	Jining	Jian Yang
162	The People’s Hospital Feixian	Eastern China	Shandong	Linyi	Honghua Deng
163	Tangshan City Fengrun District People’s Hospital	Northern China	Hebei	Tangshan	Lin Wang
164	Qian’an People’s Hospital	Northern China	Hebei	Tangshan	Yuheng Yang
165	Yuzhong County People’s Hospital	Northwest China	Gansu	Lanzhou	Xiaowei Peng
166	Baiyin Cite Center Hospital	Northwest China	Gansu	Baiyin	Fang Zhao
167	Mingguang People’s Hospital	Eastern China	Anhui	Chuzhou	Yong Li
168	Xihua County People’s Hospital	Central China	Henan	Zhoukou	Chuntong Wang
169	Zhalantun People’s Hospital	Northern China	Inner Mongolia	Hulunbeier	Yuhua Zhu
170	Fengrun District Second People’s Hospital	Northern China	Hebei	Tangshan	Jingshan Zhao
171	Zhangping City Hospital	Eastern China	Fujian	Longyan	Jinxing Yi
172	The Eight Affiliated Hospital, Sun Yat-sen University	Southern China	Guangdong	Guangzhou	Nan Jia
173	The Second Affiliated Hospital of Qiqihar Medical Hospital	Northeast China	Heilongjiang	Qiqihar	Yanli Wang
174	Fuqing Cite Hospital	Eastern China	Fujian	Fuqing	Ping Chen
175	Wuhan University of Science and Technology Hospital	Central China	Hubei	Wuhan	Jing Hu
176	Baotou City Center Hospital	Northern China	Inner Mongolia	Baotou	Ruiping Zhao
177	Shanghai Jiading District Center Hospital	Eastern China	Shanghai	Shanghai	Xia Chen

178	Shanghai Jiangwan Hospital	Eastern China	Shanghai	Shanghai	Aiping Li
179	Datong City Second People’s Hospital	Northern China	Shanxi	Datong	Xiaoqin Zhang
180	Shouyang County People’s Hospital	Northern China	Shanxi	Jinzhong	Buqiang Zhang
181	Xishan Coal Electricity Worker Ceneral Hospital	Northern China	Shanxi	Taiyuan	Suyan Zhou
182	Xixian People’s Hospital	Northern China	Shanxi	Linfen	Wenjun Yuan
183	Binyang People’s Hospital	Southern China	Guangxi	Nanning	Fudong Gan
184	Deqing People’s Hospital	Eastern China	Zhejiang	Huzhou	Fangfang Huang
185	Xinmi people’s hospital	Central China	Henan	Zhengzhou	Xiaolei Li
186	Dongguan Changping hospital	Southern China	Guangdong	Dongguan	Haiyun Lin
187	Gongyi people’s hospital	Central China	Henan	Zhengzhou	Tianmin Du
188	Ye County people’s hospital	Central China	Henan	Pingdingshan	Jie Yang
189	The second people’s hospital of Mengcheng	Eastern China	Anhui	Bozhou	Pengfei Zhang
190	Nanpi People’s Hospital	Northern China	Hebei	Cangzhou	Hui Dong
191	Shimen People’s Hospital	Central China	Hunan	Changde	Chuanliang Liang
192	Tieli People’s Hospital	Northeast China	Heilongjiang	Yichun	Yanbo Niu
193	Sihui People’s Hospital	Southern China	Guangdong	Zhaoqing	Yuehua Huang
194	Chest Hospital of Xinjiang Uygur Autonomous Region	Northwest China	Xinjiang	Urumchi	Dongsheng Chai
195	Beian First People’s Hospital	Northeast China	Heilongjiang	Heihe	Dongyan Li
196	Zunhua People’s Hospital	Northern China	Hebei	Tangshan	Xiaoli Yang
197	Lujiang People’s Hospital	Eastern China	Anhui	Hefei	Qichun Wang
198	Qinyang People’s Hospital	Central China	Henan	Jiaozuo	Xiaowen Ma

199	Longmen People's Hospital	Southern China	Guangdong	Huizhou	Yingchao Luo
200	Quyang Renji Hospital	Northern China	Hebei	Baoding	Congliang Zhang
201	Nenjiang People's Hospital	Northeast China	Heilongjiang	Heihe	Shuhua Zhang
202	Longjiang First People's Hospital	Northeast China	Heilongjiang	Qiqihar	Yuhuan Shi
203	Li County Hospital of Traditional Chinese Medicine	Central China	Hunan	Changde	Songbai Li
204	Luan County People's Hospital	Northern China	Hebei	Tangshan	Guo Li
205	Xinjin County Hospital of Traditional Chinese Medicine	Northwest China	Sichuan	Chengdu	Yingbi Su
206	Yulong Hospital	Southwest China	Yunnan	Lijiang	Zeyuan He
207	Yuncheng Hospital	Eastern China	Shandong	Heze	Jinglan Diao
208	Hepu People's Hospital	Southern China	Guangxi	Beihai	Meisheng Lai
209	Duzishan Petrochemical Hospital	Northwest China	Xinjiang	Karamay	Shuqiu Qu
210	Guiding People's Hospital	Southwest China	Guizhou	Qinan	Guoduo Chen
211	People's Hospital of Rongchang District	Southwest China	Chongqing	Chongqing	Jie Chen
212	Ningbo First Hospital	Eastern China	Zhejiang	Ningbo	Huimin Chu
213	Ledong Second People's Hospital	Southern China	Hainan	Ledong	Xiufeng Chen
214	Guang'an People's Hospital	Southwest China	Sichuan	Guang'an	Tian Tuo
215	Linfen People's Hospital	Northern China	Shanxi	Linfen	Junping Deng
216	People's Hospital of Bozhou District	Southwest China	Guizhou	Zunyi	Shengyong Chen
217	Dianjiang People's Hospital	Southwest China	Chongqing	Chongqing	Yang Yu
218	First Affiliated Hospital of Harbin Medical University	Northeast China	Heilongjiang	Harbin	Yue Li

219	Haidong Ping’an District Hospital of Traditional Chinese Medicine	Northwest China	Qinghai	Haidong	Guoqin Xin
220	Ningjin People’s Hospital	Eastern China	Shandong	Dezhou	Tao Zhang
221	Yutian Hospital	Northern China	Hebei	Tangshan	Xiaoyun Feng
222	Yanting People’s Hospital	Southwest China	Sichuan	Mianyang	Mingcheng Bai
223	The Fourth Affiliated Hospital Zhejiang University School of Medicine	Eastern China	Zhejiang	Yiwu	Shudong Xia
224	Zhongda Hospital, Southeast University (Jiangbei)	Eastern China	Jiangsu	Nanjing	Jiayi Tong
225	Wuxi Xishan People’s Hospital	Eastern China	Jiangsu	Wuxi	Xudong Li
226	Dongfeng Hospital	Northeast China	Jilin	Liaoyuan	Wei Liu
227	Zhijin People’s Hospital	Southwest China	Guizhou	Bijie	Zhongshan Wang
228	Huaiyang People’s Hospital	Central China	Henan	Zhoukou	Li Wei
229	Suizhou Central Hospital	Central China	Hubei	Suizhou	Fengwei Li
230	Tonglu First People’s Hospital	Eastern China	Zhejiang	Hangzhou	Xiaolan Li
231	Xiantao First People’s Hospital	Central China	Hubei	Xiantao	Dongmei Zhu
232	Honghu People’s Hospital	Central China	Hubei	Jingzhou	Hong Liu
233	Affilioted Hospital of North Sichuan Medical College	Northwest China	Sichuan	Nanchong	Zhan Lv
234	Guangyuan Central Hospital	Northwest China	Sichuan	Guangyuan	Bing Fu
235	Dazhou Central Hospital	Northwest China	Sichuan	Dazhou	Yong Guo
236	Nanchong Central Hospital	Northwest China	Sichuan	Nanchong	Tao Liu
